# Comparative hemocompatibility assessment of fibrin/heparin-coated and PMEA-coated flow diverters

**DOI:** 10.1007/s10856-026-07087-2

**Published:** 2026-06-13

**Authors:** Huan Wei, Tomáš Riedel, Zuzana Riedelová, Melanie Wolf, Christian Schlensak, Meltem Avci-Adali

**Affiliations:** 1 Department of Thoracic and Cardiovascular Surgery, University Hospital Tuebingen, Tuebingen, Germany; 2 Department of Chemistry and Physics of Surfaces and Biointerfaces, Institute of Macromolecular Chemistry, Czech Academy of Sciences, Prague 6, Czechia

## Abstract

Flow diverters play an important role in the endovascular treatment of intracranial aneurysms; however, their use can be associated with thromboembolic complications. Thus, in recent years, flow diverters with surface coatings have been developed to prevent thrombus-related complications. In this study, the hemocompatibility of the flow diverters FRED X with poly(2-methoxyethyl acrylate) (PMEA) coating and the DERIVO 2heal with fibrin/heparin coating, as well as the uncoated DERIVO 2 flow diverter, was evaluated using an in vitro blood circulation model. Using enzyme-linked immunosorbent assay (ELISA), biomarkers of thrombocytes (β-thromboglobulin (β-TG)), coagulation (thrombin-antithrombin complex (TAT)), complement system (SC5b-9), and inflammation (PMN elastase) activation were analyzed. Further analyses comprised thrombogenicity assessment by scanning electron microscopy and measurement of blood cell counts and hemolysis. The uncoated DERIVO 2 flow diverter exhibited increased coagulation and platelet activation, accompanied by enhanced adhesion of platelets to the blood-contacting surfaces. In contrast, both the FRED X and the DERIVO 2-heal flow diverter could effectively reduce the coagulation (TAT) and platelet activation (β-TG) and prevent the adhesion of platelets. Notably, DERIVO 2heal flow diverter induced significantly lower activation of the complement system (SC5b-9) and reduced inflammatory response (PMN elastase) compared to FRED X flow diverter. These findings indicate the improved hemocompatibility of the fibrin/heparin-coated DERIVO 2heal flow diverter and its potential to reduce thromboinflammatory complications.

**Graphical Abstract:**

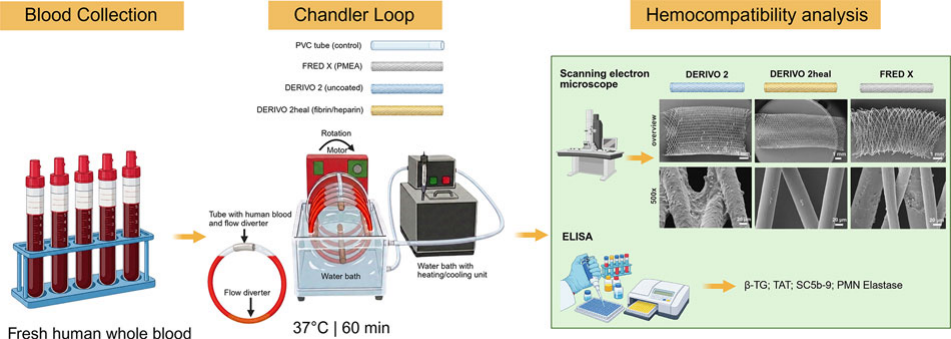

## Introduction

Intracranial aneurysms are localized dilations of cerebral vessels and are typically asymptomatic; however, rupture can lead to subarachnoid hemorrhage (SAH), a form of hemorrhagic stroke accounting for approximately 5% of all strokes worldwide, with most cases resulting from ruptured intracranial aneurysms [1]. In general, the incidence of SAH varies by gender, age group, and geographical region [2]. The overall incidence of aneurysmal SAH (aSAH) is estimated at approximately 6.67 per 100,000 person-years, with women overall being at higher risk than men, particularly older women in Japan and Europe [3, 4].

The incidence of SAH increased significantly from 1990 to 2021, whereas the number of deaths and disability‑adjusted life‑years (DALYs) attributed to SAH declined over time. Additionally, both age-standardized mortality rates (ASMRs) and age-standardized disability-adjusted life-year rates (ASDRs) demonstrated a downward trend. These changes likely reflect advances in medical care, particularly improvements in interventional therapies for cerebrovascular diseases since 1990 [2].

The first surgical treatment of aneurysms dates back to the 19th century. However, the first aneurysm clipping procedure was performed in 1937 and subsequently remained the gold standard for aneurysm treatment for a long time thereafter [5, 6]. The progress in interventional techniques for managing intracranial aneurysms, together with the findings from the International Subarachnoid Hemorrhage Trial, has prompted a paradigm shift from open surgical approaches toward endovascular management [7]. Endovascular treatment strategies include coil embolization and the use of densely woven flow diverters. By using high-metal-density mesh structure, flow diverters alter intra-aneurysmal hemodynamics by modifying inflow and outflow jets, thereby promoting thrombosis within the aneurysm sac, preventing further expansion, and effectively reducing the risk of rupture [8–10]. Following implantation, neointimal overgrowth develops along the device surface, covering the stent struts and thereby reconstructing the parent artery while eliminating the aneurysm–parent vessel interface [9]. Compared with other neurovascular implants, such as laser-cut stents, flow diverters introduce a substantially larger amount of foreign material into the vessel lumen due to their dense wire mesh structure, which can provoke a foreign body response against the implant. This response can lead to serious complications, including ischemic stroke due to device occlusion, as well as thromboembolic and hemorrhagic events [11–13].

To improve the hemocompatibility of blood-contacting implants and thereby reduce device-related potential complications, extensive research has focused on the development of surface coatings using various strategies [14, 15]. Consequently, medical device manufacturers have recognized the substantial potential of such technologies to improve patient safety, leading to the application of biocompatible coatings on flow-diverters that were previously uncoated. Medtronic (Minneapolis, MN, USA) employs a phosphorylcholine (component of outer cell membranes)-based coating for the Pipeline Flex Shield Embolization Device, which has been shown to reduce the thrombogenicity of the flow diverter [13, 16]. Phenox GmbH (Bochum, Germany) achieves the same effect with its hydrophilic polymer coating (pHPC®) technology on its flow diverters p48 MW and p64 MW by mimicking cell surface glycocalyx molecules [17]. In contrast to cell membrane–mimicking strategies, Terumo Neuro’s X-technology (Aliso Viejo, California, CA, USA) is based on a covalently bound nanoscale layer of poly(2-methoxyethyl acrylate) (PMEA), a hydrophilic polymer that forms a highly hydrated interface when exposed to aqueous environments [18]. The hemocompatibility of PMEA-based coatings has been attributed to their ability to maintain a stable hydration layer at the blood–material interface, which limits nonspecific protein adsorption and subsequent thrombotic reactions [13, 19, 20]. Another medical technology manufacturer of flow diverters, Acandis GmbH, applies natural components for its coating technology HEAL. The HEAL coating consists of a fully polymerized fibrin network and covalently bound heparin molecules [21]. Due to this composition, the coating exhibits antithrombogenic and anti-inflammatory properties while simultaneously promoting endothelialization [21–23].

In this study, the hemocompatibility of the HEAL-coated DERIVO 2 flow diverter (DERIVO 2heal, Acandis GmbH Pforzheim, Germany) was evaluated under flow conditions with fresh human blood and compared with the flow redirection endoluminal device (FRED) X (Terumo Neuro) and with the flow diverter without surface coating, the DERIVO 2 (Acandis GmbH Pforzheim, Germany).

## Materials and methods

### Flow diverter

Commercially available flow diverter for the treatment of aneurysms, Terumo Neuro (MicroVention) FRED X 27 (4.5 ×25 mm, Aliso Viejo, CA, USA) and DERIVO 2 and DERIVO 2heal (both 4.5 ×20 mm, Acandis GmbH, Pforzheim, Germany) were tested.

### Blood collection

Human whole blood was collected from five healthy, non-medicated donors by venipuncture. Blood was drawn into 9 mL monovettes (Sarstedt, Nümbrecht, Germany) prefilled with sodium heparin 25000 (1.5 IU/ml, LEO Pharma GmbH, Neu-Isenburg, Germany). Blood sampling procedures were approved by the Ethics Committee of the University of Tuebingen, and written informed consent was obtained from all participants.

### Dynamic incubation of flow diverters with human blood in the Chandler loop model

Chandler loop model [24] was employed to simulate blood circulation and assess the hemocompatibility of the flow diverters at 37 °C. Each device was positioned within a 50 cm long PVC tube (1/4” x 1/16”, RAUMEDIC, Helmbrechts, Germany). A cable tie placed proximal to the inserted flow diverter ensured the position of the flow diverter during circulation. The tubes were then filled with 12 ml of fresh human whole blood each, closed into loops, and rotated at 30 rpm for 60 min. Baseline measurements were obtained immediately after blood collection, without dynamic incubation. Identically prepared empty tubes containing the same blood volume served as negative controls. Following 60 min of dynamic incubation, blood plasma was generated by centrifugation, and each sample was immediately shock-frozen using liquid nitrogen and stored at −80 °C until analysis.

### Analysis of hemocompatibility markers

Cellular blood parameters, platelet, erythrocyte, and leukocyte counts, as well as hematocrit and hemoglobin levels, were determined using an automated hematology analyzer (Micros 60, ABX Hematology, Montpellier, France).

Hemolysis was assessed using the cyanmethemoglobin method through determination of free hemoglobin in the plasma by measuring the absorption at 540 and 680 nm spectrophotometrically (EPOCH2, BioTek, Santa Clara, CA, USA) .

Plasma samples were further evaluated by immunochemical analysis using enzyme-linked immunosorbent assays (ELISAs) to quantify markers of coagulation (thrombin-antithrombin III complex (TAT), Siemens Healthcare Diagnostics Products GmbH, Marburg, Germany), platelet (β-thromboglobulin (β-TG), Diagnostica Stago S.A.S, Asnières sur Seine, France), complement system (SC5b-9, Quidel, San Diego, CA, USA), and leukocytes (PMN-Elastase-α1-PI complex (PMN-elastase), Demeditec Diagnostics GmbH, Kiel, Germany) activation.

### Scanning electron microscopy (SEM)

Following incubation with human blood in the Chandler loop model, the flow diverters were washed three times with 200 µl Dulbecco’s phosphate-buffered saline (DPBS, Sigma-Aldrich, Munich, Germany). Subsequently, the samples were fixed in DPBS containing 2% glutaraldehyde (Serva, Heidelberg, Germany) for 70 min at RT and then washed again with DPBS for 15 min at RT. Dehydration was performed using a graded ethanol series (40–100% ethanol; Merck-Millipore, Darmstadt, Germany), with stepwise incubations of 15 min each at RT. The samples were dried using a critical point drier (Polaron E3100, Quorum Technologies Ltd, East Sussex, United Kingdom), sputter-coated with gold, and subsequently analyzed by SEM (EVO LS 10, Carl Zeiss, Oberkochen, Germany).

### Statistical analysis

Data are presented as mean + standard deviation (SD). Statistical analyses were performed using GraphPad PRISM software version 10.0.2 (GraphPad Software, Inc., San Diego, US). Potential outliers were identified using Grubbs’ test and excluded prior to statistical analysis (α = 0.05). One-way repeated measures ANOVA followed by Tukey´s multiple comparison test was used to evaluate differences between groups. A p-value of ≤ 0.05 was considered statistically significant.

## Results

### Analysis of blood cell counts and hemolysis

**Fig. 1:**
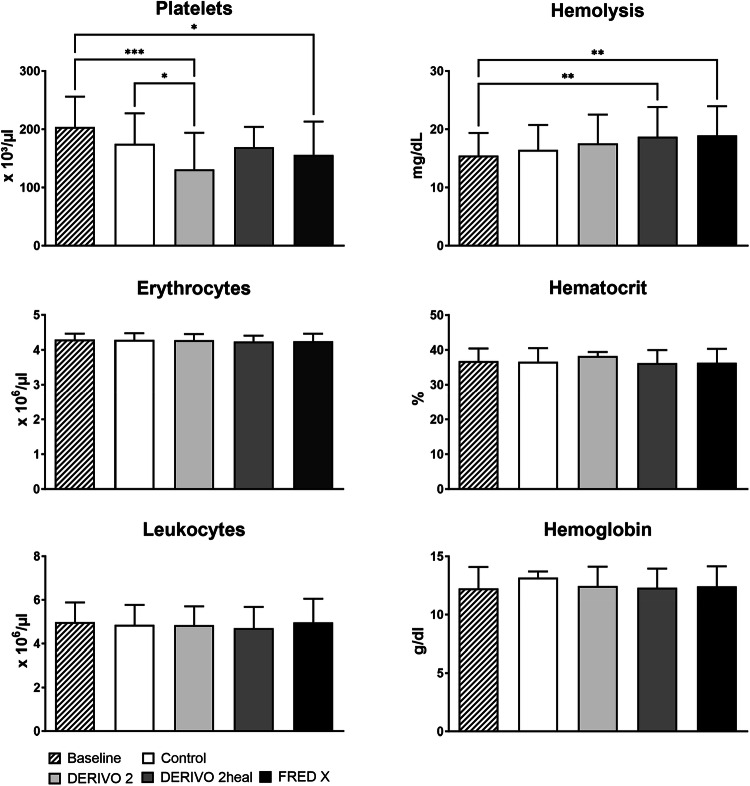
Analysis of blood cell counts, hemolysis, hematocrit, and hemoglobin levels. Test devices (DERIVO 2, DERIVO 2heal, and FRED X) were incubated in a Chandler loop model with fresh human blood for 60 min. As a control, levels were determined immediately after the collection of blood (baseline) and after the incubation of blood in the test system without test material (control). Platelet, erythrocyte, and leukocyte numbers (*n* = 5), hemoglobin (*n* = 5, except control group *n* = 4) and hematocrit levels (*n* = 5, except DERIVO 2 group *n* = 4), and hemolysis (*n* = 4) were analyzed. Results are presented as mean + SD. *p ≤ 0.05, **p ≤ 0.01, ***p ≤ 0.001

The numbers of thrombocytes, erythrocytes, leukocytes, hemolysis, and the levels of hemoglobin and hematocrit were determined after 60 min of blood contact in the in vitro rotation model (Fig. 1). After 60 min of incubation, the uncoated DERIVO 2 flow diverter resulted in a significant reduction in platelet counts compared with both baseline and the control tube, indicating an adhesion of platelets to the test material. Blood samples incubated with the FRED X flow diverter also showed a significant decrease in platelet counts relative to baseline; however, no significant difference was observed compared with the control group. Leukocyte and erythrocyte counts as well as hemoglobin and hematocrit levels remained unchanged across all groups. Only a slight increase in hemolysis was detected in samples incubated with the DERIVO 2heal and FRED X flow diverter groups compared with baseline after the 60-minute incubation. However, free hemoglobin levels did not differ significantly from those measured in the empty control tube, indicating the compatibility of the devices with erythrocytes.

### Analysis of the thrombogenicity using SEM analysis

**Fig. 2:**
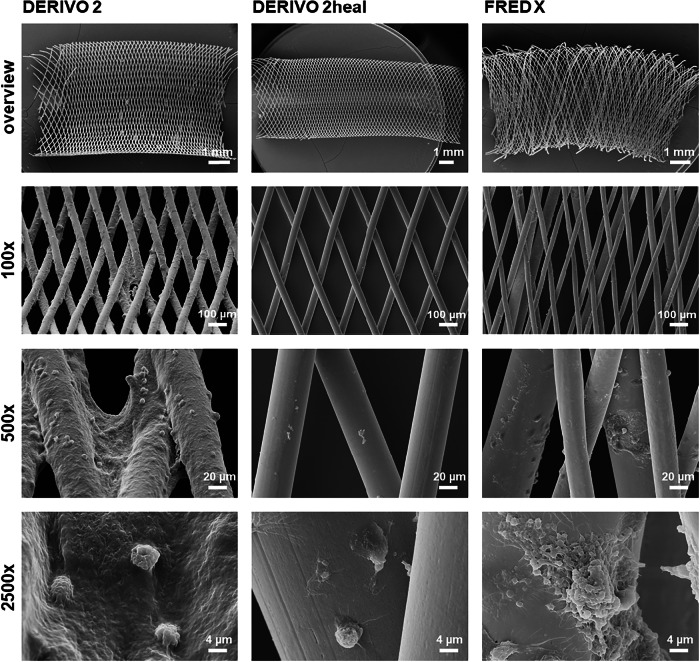
Scanning electron microscopy (SEM) images of the surface of the tested flow diverters. All devices were incubated for 60 min with fresh whole human blood in a Chandler loop model. Representative SEM images from a single donor are shown (*n* = 5).

SEM analyses showed increased adhesion of platelets and fibrin generation on the surface of the uncoated DERIVO 2 flow diverters (Fig. 2). On the surface of DERIVO 2heal and FRED X flow diverters, fibrin formation and the adhesion of thrombocytes were observed only in isolated areas. The majority of the stent surface remained without any remarkable deposits.

### Analysis of platelet and coagulation activation

**Fig. 3:**
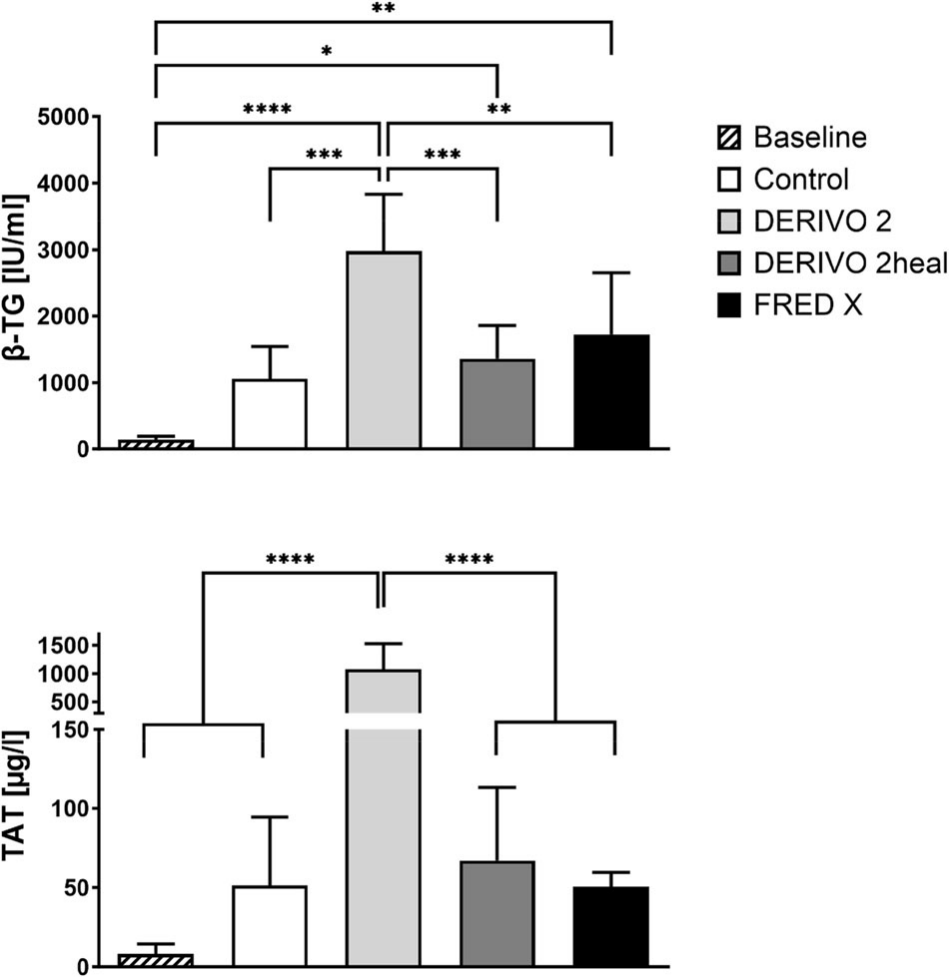
Assessment of platelet and coagulation activation. Fresh human blood was exposed to the test devices (DERIVO 2, DERIVO 2heal, and FRED X) for 60 min in a Chandler loop model. Reference measurements were obtained immediately after blood withdrawal (baseline) and following incubation in the loop system without device exposure (control). Platelet activation was evaluated by measuring β-thromboglobulin (β-TG) (*n* = 5, except baseline group *n* = 4), while coagulation activation was assessed by quantification of thrombin–antithrombin (TAT) complexes (*n* = 5, except DERIVO 2 and FRED X group *n* = 4). Results are presented as mean + SD. *p ≤ 0.05, **p ≤ 0.01, ***p ≤ 0.001, ****p ≤ 0.0001

The release of β-TG from platelet granules correlates with the activation of platelets, and elevated levels of TAT complexes indicate activation of the coagulation cascade. Thus, increased β-TG and TAT levels indicate an increased risk of thrombosis. Accordingly, β-TG levels were measured to assess platelet activation, whereas TAT levels were determined to evaluate coagulation activation (Fig. 3).

The incubation of DERIVO 2 flow diverters with human blood resulted in significantly increased β-TG and TAT levels compared to DERIVO 2heal and FRED X flow diverters, the control group, and baseline, indicating an activation of thrombocytes and coagulation. Flow diverters with fibrin/heparin (DERIVO 2heal) coating or the polymer poly(2-methoxyethyl acrylate) (PMEA) coating (FRED X) prevented the activation of platelets and coagulation and showed similar β-TG and TAT levels to the control tube.

### Analysis of inflammation and complement system activation

**Fig. 4:**
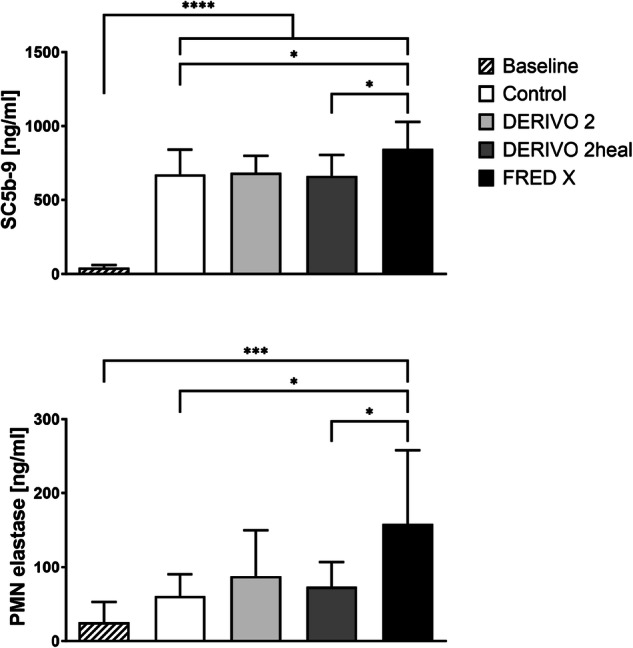
Analysis of leukocyte and complement system activation. Fresh human blood was incubated for 60 min with test devices (DERIVO 2, DERIVO 2heal, and FRED X) in a Chandler loop model. As a control, levels were determined immediately after the collection of blood (baseline) and after the incubation of blood in the test system without test material (control). The levels of the terminal complement complex SC5b-9 and the inflammation marker (PMN-elastase) were determined. Results are presented as mean + SD (*n* = 5). *p ≤ 0.05, ***p ≤ 0.001, ****p ≤ 0.0001

The terminal complement complex SC5b-9 is a highly sensitive marker of complement system activation. Therefore, elevated levels indicate activation of the complement system. In addition, activation of neutrophil granulocytes results in the release of PMN elastase, making increased PMN elastase levels indicative of leukocyte activation. In the in vitro rotation model, blood contact with the DERIVO 2heal devices resulted in significantly lower levels of SC5b-9 and PMN elastase compared with the FRED X flow diverter (Fig. 4). These findings indicate a lower potential for complement system activation and inflammatory response associated with the DERIVO 2heal surfaces compared to the FRED X flow diverter. Blood samples incubated with the DERIVO 2 and DERIVO 2heal devices exhibited SC5b-9 and PMN elastase levels comparable to those of the control group, indicating a very low immune response to the DERIVO family.

## Discussion

Exposure of blood to artificial surfaces, such as those of flow diverters, can trigger a cascade of biological responses that can ultimately activate the coagulation cascade as well as platelets [25]. The contact activation and kallikrein-kinin systems, collectively known as the plasma-contact system, play central roles in initiating coagulation, innate immunity, and inflammation [26]. Upon surface contact, Factor XII undergoes autoactivation, resulting in the generation of Factor XIIa. Activated factor FXIIa can consequently activate the complement system and FXI, leading to thrombin generation via the intrinsic coagulation pathway [27–29]. The generated thrombin can activate platelets, promote secretion of bioactive substances from granules and yield a surface for prothrombinase assembly and ultimately clot formation [29]. Therefore, the development of thromboresistant surface coatings that mitigate the risk of thrombogenic events following the implantation of flow diverters is highly desirable. Optimized surface modifications can substantially improve the biocompatibility and hemocompatibility of these devices, thereby ensuring patient safety [30].

In this study, the hemocompatibility of the fibrin/heparin-based nanocoating of the DERIVO 2heal flow diverter was investigated in comparison to the PMEA-coated FRED X flow diverter using fresh human whole blood in a Chandler loop circulation system according to the ISO 10993-4 guidelines.

Erythrocyte and leukocyte counts, as well as hemoglobin and HCT hematocrit levels, were unchanged in all flow diverter groups and comparable to the empty tube control group. In addition, no increased hemolysis was induced by the flow diverters compared with the control tube.

Following a 60 min incubation period, plasma concentrations of TAT and β-TG demonstrated a very low blood coagulation activation potential of the DERIVO 2heal flow diverter, comparable to that of the Terumo Neuro FRED X flow diverter. Compared with the control tube, incubation with the coated devices did not result in a significant reduction in platelet counts. In contrast, the uncoated DERIVO 2 flow diverter caused a significant decrease in platelet counts, indicating enhanced platelet adhesion. These findings were corroborated by SEM analysis, which revealed reduced fibrin deposition on the surfaces of the DERIVO 2heal and FRED X flow diverters compared with the uncoated DERIVO 2 flow diverter, demonstrating reduced thrombogenicity associated with the antithrombogenic coatings. Since TAT, β-TG, and platelet counts directly depict the thrombogenicity of an implant, the antithrombogenic properties of the coated flow diverters can be concluded based on the measured values and the comparison with the control group. The uncoated DERIVO 2 flow diverter exhibited the highest levels of coagulation (TAT) and platelet activation (β-TG), and the greatest reduction in platelet counts.

Both the DERIVO 2 and the earlier generation have been extensively evaluated in clinical studies for aneurysm treatment. Reported outcomes showed complete aneurysm occlusion rates between 75% [31] and 89.2% [32]. When considering adequate occlusion, defined as complete occlusion or a residual neck according to O’Kelly - Marotta grades D and C, success rates ranged from 89% [33] to 100% [34]. The first multicentric and retrospective evaluation of the HEAL-coated DERIVO 2heal in a very early follow-up at 6.6 months showed adequate occlusion of 80.7% [35]. This result is consistent with the results from previous uncoated generations of the flow diverter, which already demonstrated high occlusion rates. The comparison indicates that larger patient cohorts are required to detect a difference in occlusion rates attributable to the HEAL technology.

In patients treated with the uncoated DERIVO device, reported rates of major stroke and ischemic events ranged from 0.56% [36] to 4.2% [33]. In a more recent investigation of the DERIVO 2 device, thromboembolic complications were observed in 2.7% of cases [37]. These rates are similar to the 4.8% of minor and 0% major ischemic complications shown for the DERIVO 2heal [35]. In a small retrospective analysis, Uysal et al. revealed the safety and efficacy of the DERIVO 2heal under single antiplatelet therapy using dose-adjusted P2Y12 inhibitors with 3.8% ischemic complication rate and 91.7% complete occlusion at 14.5 months follow-up [38]. This result suggests the clinical relevance of the anti-thrombogenic effect of HEAL technology.

The uncoated FRED device showed complete occlusion rates between 55.6% [39] and 75.1% (Raymond Roy Classification) [40] and an adequate occlusion rate of 88.9% (Raymond Roy I &II) [39] at the 12-month follow-up. In comparison, the coated FRED X flow diverters depicted similar rates between 60% and 69.8% for and 86.7 and 83.9% for complete or adequate occlusion rate, respectively [39, 41]. In a direct comparison between the FRED and FRED X stroke rates were comparable with 2.86% and 3.77%, respectively [39].

Assessment of leukocyte activation is not only essential for characterizing the inflammatory response to an implant but also for evaluating its thrombogenic potential. Activated leukocytes can release procoagulant and proinflammatory mediators, which can trigger platelet activation and adhesion as well as the coagulation cascade. In general, inflammation not only initiates clot formation but also downregulates endogenous anticoagulant mechanisms and fibrinolysis [42]. In this study, incubation of blood with the DERIVO 2heal and the uncoated DERIVO flow diverter showed no statistically significant differences compared with the control group, indicating that neither device induced inflammation, as assessed by PMN elastase levels. In contrast, the FRED X device demonstrated a significant activation of leukocytes and an increased inflammatory response. Accordingly, the low inflammatory activation observed for both DERIVO 2heal and DERIVO 2 flow diverters could be advantageous compared to the FRED X diverter.

The Chandler loop investigation further revealed significantly lower levels of complement activation marker SC5b-9 for the DERIVO 2 and the DERIVO 2heal devices compared with the FRED X device. Moreover, both the uncoated and heal-coated DERIVO 2 flow diverters showed no increase in SC5b-9 levels relative to the control tube. The importance of the complement system in mediating inflammatory and thrombogenic processes by foreign materials, such as flow diverters, has been previously described in Girbas et al. [22]. Overall, low complement activation is a desirable characteristic for any permanent implant.

DERIVO 2 and FRED X flow diverter are comparable with regard to their interventional applicability; however, they differ substantially in structural design and dimensional characteristics. The FRED X flow diverter is composed of a dual-layer mesh architecture, whereas the DERIVO 2heal consists of a single braided layer. As a consequence of its dual-layer design, the FRED X is characterized by two distinct lengths that must be considered during implantation: the working length and the total length. The working length corresponds to the inner mesh layer, which is primarily responsible for the flow-diverting effect. In the present study, the FRED X devices exhibited a working length of 18 mm and a total length of 25 mm when deployed in a vessel with a diameter of 4.5 mm. For comparative purposes, a DERIVO 2heal device with a nominal length of 20 mm was selected, as this most closely approximates the working length of the FRED X. Owing to its dual-layer configuration, the FRED X is expected to present a larger effective surface area than the single-layer DERIVO 2heal, although this difference cannot be quantified precisely. In general, an increased surface area corresponds to a greater amount of implanted foreign material, which may be associated with an elevated risk of foreign body reactions and thrombogenicity.

The coating of implant surfaces with endogenous agents, such as the HEAL technology, represents a promising approach to improve the biocompatibility and hemocompatibility of blood-contacting devices. In this in vitro study, an improved hemocompatibility of the DERIVO 2heal flow diverter was again demonstrated, consistent with the findings reported by Girbas et al. [22]. Moreover, additional both in vivo and in vitro investigations have demonstrated that the HEAL coating has antithrombogenic and anti-inflammatory effects while simultaneously promoting endothelialization [21, 43].

## Conclusion

This in vitro study demonstrated that the fibrin/heparin coating (DERIVO 2heal) enhances hemocompatibility relative to the uncoated DERIVO 2 flow diverters. The coagulation and platelet activation potential of DERIVO 2heal flow diverters was comparable to that of the FREDX flow diverter, whereas the DERIVO 2heal flow diverter elicited significantly less leukocyte and complement system activation compared with the FREDX flow diverter. This may represent a substantial enhancement in performance, owing to a potentially lower inflammatory response and improved healing. Consequently, together with the potential for enhanced adhesion and proliferation of endothelial cells, the HEAL coating of DERIVO 2 flow diverters could constitute a next-generation coating for an improved treatment option for patients with intracranial aneurysms.

To obtain even more meaningful results regarding the comparability of HEAL- and X-technology, these in vitro findings should be validated in relevant in vivo models, with particular emphasis on endothelialization, neointimal formation, and inflammatory response under physiological flow conditions. Additionally, comparative clinical studies are warranted to assess whether the reduced leukocyte and complement activation observed with the DERIVO 2heal translates into lower thromboembolic and inflammatory complication rates in patients to fully define its clinical benefit and potential role as a next-generation flow diverter technology.

## References

[1] Xu Z. Intracranial aneurysms: pathology, genetics, and molecular mechanisms. Neuromol Med. 2019;21:325–43. 10.1007/s12017-019-08537-7 PMC6829066 31055715

[2] Lv B. Epidemiological trends of subarachnoid hemorrhage at global, regional, and national level: a trend analysis study from 1990 to 2021. Mil Med Res. 2024;11:46. 38992778 10.1186/s40779-024-00551-6 PMC11241879

[3] Hughes JD. Estimating the global incidence of aneurysmal subarachnoid hemorrhage: a systematic review for central nervous system vascular lesions and meta-analysis of ruptured aneurysms. World Neurosurg. 2018;115:430–47.e7. 29649643 10.1016/j.wneu.2018.03.220

[4] Etminan N. Worldwide incidence of aneurysmal subarachnoid hemorrhage according to region, time period, blood pressure, and smoking prevalence in the population: a systematic review and meta-analysis. JAMA Neurol. 2019;76:588–97. 30659573 10.1001/jamaneurol.2019.0006 PMC6515606

[5] Dandy WE. Intracranial aneurysm of the internal carotid artery: cured by operation. Ann Surg. 1938;107:654–9. 17857170 10.1097/00000658-193805000-00003 PMC1386933

[6] Belavadi R. Surgical clipping versus endovascular coiling in the management of intracranial aneurysms. Cureus. 2021;13:20478. 10.7759/cureus.20478 PMC8760002 35047297

[7] Molyneux A. International Subarachnoid Aneurysm Trial (ISAT) of neurosurgical clipping versus endovascular coiling in 2143 patients with ruptured intracranial aneurysms: a randomised trial. Lancet. 2002;360:1267–74. 12414200 10.1016/s0140-6736(02)11314-6

[8] Seshadhri S. Impact of stents and flow diverters on hemodynamics in idealized aneurysm models. J Biomech Eng. 2011;133:071005. 21823744 10.1115/1.4004410

[9] Kim S. Computational study of hemodynamic changes induced by overlapping and compacting of stents and flow diverter in cerebral aneurysms. Front Neurol. 2021;12:705841. 34408723 10.3389/fneur.2021.705841 PMC8365227

[10] Alderazi YJ. Flow diverters for intracranial aneurysms. Stroke Res Treat. 2014;2014:415653–12. 24967131 10.1155/2014/415653 PMC4054970

[11] Arrese I. Flow-diverter devices for intracranial aneurysms: systematic review and meta-analysis. Neurosurgery. 2013;73:193–9. 23624409 10.1227/01.neu.0000430297.17961.f1

[12] Fiorella D. Very late thrombosis of a pipeline embolization device construct: case report. Neurosurgery. 2010;67:onsE313–onsE314. 20679914 10.1227/01.NEU.0000383875.08681.23

[13] White TG. Flow diverter surface modifications for aneurysm treatment: A review of the mechanisms and data behind existing technologies. Inter Neuroradiol. 2026;32:109–25. 10.1177/15910199231207550 PMC12852632 37899636

[14] Raikar AS. Surface engineering of bioactive coatings for improved stent hemocompatibility: a comprehensive review. Materials. 2023;16:6940. 37959540 10.3390/ma16216940 PMC10650382

[15] Schumacher AL. Development and evaluation of a nanometer-scale hemocompatible and antithrombotic coating technology platform for commercial intracranial stents and flow diverters. ACS Appl Nano Mater. 2018;1:344–54.

[16] Starke RM. Preclinical safety and efficacy evaluation of the Pipeline Vantage Embolization Device with Shield Technology. J Neurointerv Surg. 2020;12:981–6. 32487767 10.1136/neurintsurg-2020-016043 PMC7509525

[17] Bilgin C. Phenox HPC and Phenox flow modulation devices for the endovascular treatment of intracranial aneurysms: a systematic review and meta-analysis. J Neurointerv Surg. 2024;16:706–14. 37536930 10.1136/jnis-2023-020514

[18] Abbas R. First United States multicenter experience with the new-generation FRED X surface-modified flow diversion stent: feasibility, safety, and short-term efficacy. J Neurosurg. 2024;140:1054–63. 37856406 10.3171/2023.7.JNS23966

[19] Yoshizawa K. Poly(2-methoxyethyl acrylate) (PMEA) improves the thromboresistance of FRED flow diverters: a thrombogenic evaluation of flow diverters with human blood under flow conditions. J Neurointerv Surg. 2023;15:1001–6. 36180206 10.1136/jnis-2022-019248 PMC10511968

[20] Tanaka M. Design of biocompatible and biodegradable polymers based on intermediate water concept. Polym J. 2015;47:114–21.

[21] Muhl-Benninghaus R. Vascular response on a novel fibrin-based coated flow diverter. Cardiovasc Interv Radio. 2022;45:236–43. 10.1007/s00270-021-03007-9 PMC8807434 34913987

[22] Girbas MG. Comparison of the hemocompatibility of neurovascular flow diverters with anti-thrombogenic coatings. J Sci Adv Mater Devices. 2024;9:100666.

[23] Filova E. Improved adhesion and differentiation of endothelial cells on surface-attached fibrin structures containing extracellular matrix proteins. J Biomed Mater Res A. 2014;102:698–712. 23723042 10.1002/jbm.a.34733

[24] Sinn S. A novel in vitro model for preclinical testing of the hemocompatibility of intravascular stents according to ISO 10993-4. J Mater Sci Mater Med. 2011;22:1521–8. 21604053 10.1007/s10856-011-4335-2

[25] Kizhakkedathu JN, Conway EM. Biomaterial and cellular implants: foreign surfaces where immunity and coagulation meet. Blood. 2022;139:1987–98. 34415324 10.1182/blood.2020007209

[26] Schmaier AH. The contact activation and kallikrein/kinin systems: pathophysiologic and physiologic activities. J Thromb Haemost. 2016;14:28–39. 26565070 10.1111/jth.13194

[27] Maas C, Oschatz C, Renne T. The plasma contact system 2.0. Semin Thromb Hemost. 2011;37:375–81. 21805443 10.1055/s-0031-1276586

[28] Irmscher S. Kallikrein Cleaves C3 and Activates Complement. J Innate Immun. 2018;10:94–105. 29237166 10.1159/000484257 PMC6757171

[29] Hoffman M, Monroe DM. 3rd, A cell-based model of hemostasis. Thromb Haemost. 2001;85:958–65. 11434702

[30] Manivasagam VK. Surface modification strategies to improve titanium hemocompatibility: a comprehensive review. Mater Adv. 2021;2:5824–42. 34671743 10.1039/d1ma00367d PMC8451052

[31] Piano M. Long-term follow-up of the Derivo® Embolization Device (DED®) for intracranial aneurysms: the Italian Multicentric Registry. J Neurosurgical Sci. 2021;65:361–8. 10.23736/S0390-5616.21.05300-5 33879762

[32] Trivelato FP. Derivo embolization device for the treatment of intracranial aneurysms: a multicenter study of 183 aneurysms. Stroke. 2019;50:2351–8. 31288675 10.1161/STROKEAHA.119.025407

[33] Taschner CA. Derivo embolization device in the treatment of unruptured intracranial aneurysms: a prospective multicenter study. J NeuroInterventional Surg. 2021;13:541–6. 10.1136/neurintsurg-2020-016303 PMC8142444 32900908

[34] Goertz L. Improved occlusion rate of intracranial aneurysms treated with the Derivo embolization device: one-year clinical and angiographic follow-up in a multicenter study. World Neurosurg. 2019;126:e1503–e1509. 30910748 10.1016/j.wneu.2019.03.137

[35] Schwab R. The DERIVO 2 heal embolization device in the treatment of ruptured and unruptured intracranial aneurysms: a retrospective multicenter study. Clin Neuroradiol. 2025;35:25–34. 39172220 10.1007/s00062-024-01446-8 PMC11832578

[36] Lourenço GC. Endovascular treatment of intracranial aneurysms using the Derivo Embolization Device: a multicenter experience. J Neurointerv Surg. 2023;15(8):776–780. 10.1136/neurintsurg-2022-018648 10.1136/neurintsurg-2022-018648 35705359

[37] Thormann M, The DERIVO® 2 Embolization Device in the treatment of ruptured and unruptured intracranial aneurysms: a multicenter analysis. Int Neuroradiol. 2024;30(5):672–678. 10.1177/15910199221142643 PMC11569461 36567499

[38] Uysal A. Initial experience with the derivo 2-heal flow diverter under standard or reduced-dose single antiplatelet therapy. Am J Neuroradiol. 2024;45:1038–43. 39025640 10.3174/ajnr.A8292 PMC11383417

[39] Roy JM. Comparative analysis of safety and efficacy of flow diversion with and without surface modification technology, FRED-X, FRED, PED shield and PED in 386 patients: A single center experience with systematic review and network meta-analysis. J Neurological Sci. 2025;468:123336. 10.1016/j.jns.2024.123336 39700780

[40] Waqas M. Flow redirection endoluminal device (FRED) for treatment of intracranial aneurysms: a systematic review. Int Neuroradiol. 2022;28:347–57. 10.1177/15910199211027991 PMC9185102 34192977

[41] Mortezaei, A. FRED X flow diversion stent for intracranial aneurysms: a systematic review and meta-analysis. Neuroradiology 2025. 10.1007/s00234-025-03755-2. Epub ahead of print. PMID: 40970960 10.1007/s00234-025-03755-2 40970960

[42] Swystun LL, Liaw PC. The role of leukocytes in thrombosis. Blood. 2016;128:753–62. 27354721 10.1182/blood-2016-05-718114

[43] Kaplan O. Low-thrombogenic fibrin-heparin coating promotes in vitro endothelialization. J Biomed Mater Res Part A. 2017;105:2995–3005. 10.1002/jbm.a.36152 28646555

